# Diagnostic and therapeutic potential of resolvin D1 in Guillain–Barré syndrome

**DOI:** 10.1016/j.jare.2025.10.073

**Published:** 2025-11-02

**Authors:** Ying Wang, Shan Liu, Mingqin Zhu, Qingxiang Zhang, Hui Sun, Kangding Liu, Jie Zhu

**Affiliations:** a Department of Neurology, Neuroscience Center, The First Hospital of Jilin University, Changchun 130000, China; b Department of Nuclear Medicine, The Second Hospital of Jilin University, Changchun 130000, China; c Institute of Neurological Diseases, Department of Neurology, The First Affiliated Hospital of Henan University, Kaifeng 457000, China; d Department of Neurobiology, Care Sciences & Society, Karolinska Institute, Karolinska University Hospital, Solna SE-171 64, Sweden

**Keywords:** Guillain–Barré syndrome, Experimental autoimmune neuritis, Neuroimmune diseases, Inflammation resolution, Resolvin D1

## Abstract

**Introduction:**

Most neuroimmune diseases are incurable and require lifelong immunotherapy, whereas Guillain–Barré syndrome (GBS), a peripheral nervous system autoimmune disorder, has a relatively favorable prognosis. Investigating the molecular disparities underlying recovery between GBS and other neuroimmune diseases may facilitate the development of innovative therapeutic strategies.

**Objectives:**

This study explored the diagnostic and therapeutic potential of resolvin D1 (RvD1), a specialized pro-resolving mediator that promotes inflammation resolution and tissue regeneration, in GBS and its animal model experimental autoimmune neuritis (EAN).

**Methods:**

Serum RvD1 levels were measured in 218 participants from two cohorts, including healthy controls (HCs), GBS patients, and those with multiple sclerosis (MS), neuromyelitis optica spectrum disorder (NMOSD), or stroke. The diagnostic accuracy was evaluated via the area under the receiver operating characteristic (ROC) curve (AUC). RvD1 was administered to EAN and experimental autoimmune encephalomyelitis (EAE, an MS animal model) to assess its therapeutic efficacy.

**Results:**

Serum RvD1 was elevated in GBS patients, demonstrating high diagnostic accuracy in differentiating GBS patients from HCs (cohort 1: AUC 0.72; cohort 2: AUC 0.93), MS/NMOSD patients (cohort 1: AUC 0.83), and stroke patients (cohort 1: AUC 0.68). The RvD1 level continued to increase as GBS progressed and was positively correlated with disease severity, ventilator dependence, and axonal neuropathy. In EAN, RvD1 and its synthetase/receptor were upregulated in serum, spleen, and sciatic nerve, exhibiting tissue-specific inflammation resolution patterns across disease phases. RvD1 treatment promoted inflammation resolution in EAN, as evidenced by attenuated disease severity and autoimmune responses, with its therapeutic effect was translatable to EAE.

**Conclusion:**

RvD1 may serve as a biomarker for both diagnosing GBS and monitoring disease severity. It represents a potential immunotherapeutic agent for GBS and other neuroimmune disorders. Elucidating the molecules associated with recovery in patients with GBS may facilitate the discovery of novel therapeutic strategies for neuroimmune disorders.

## Highlights

• Serum RvD1 distinguishes GBS from HCs and other neuroimmune diseases with good diagnostic accuracy.
• Serum RvD1 levels correlate with GBS severity, ventilator dependence, and axonal neuropathy.
• RvD1 and its synthetase/receptor are upregulated in EAN with tissue-specific dynamics.
• RvD1 treatment alleviates EAN and EAE, indicating its therapeutic potential for neuroimmune diseases.

## Graphical abstract

In brief, Wang et al. reported that serum RvD1 has potential as a diagnostic biomarker for GBS, as it differentiates patients with GBS from healthy controls and those with other CNS diseases with high sensitivity and specificity. Moreover, RvD1 may represent a promising immunotherapeutic agent for treating neuroimmune disorders, as its administration attenuates disease severity and autoimmune responses in EAN, with therapeutic effects that could also translate to EAE. The figure was created via BioRender (https://biorender.com).

**Figure:**
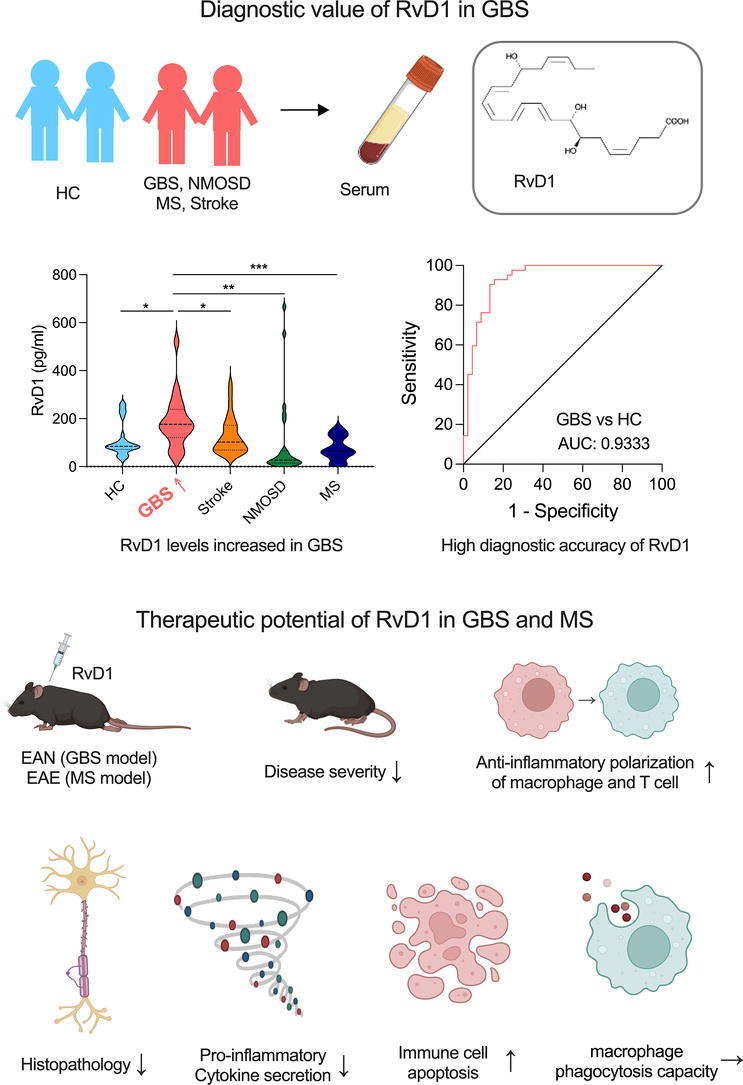

## Introduction

Neuroimmune diseases encompass a spectrum of disorders in which the immune system abnormally attacks the nervous system, leading to damage to nerve tissues and impaired functions. Neuroimmune diseases typically follow a chronic course characterized by recurrent relapses or persistent progression, requiring most patients to undergo lifelong immunotherapy [1,2]. Therefore, new therapeutic strategies are urgently needed. Guillain–Barré syndrome (GBS) is an autoimmune disorder that primarily affects the peripheral nervous system [3,4]. Although the pathogenesis of GBS shares commonalities with that of other neuroimmune diseases, such as aberrant activation of autoreactive T cells and abnormal secretion of autoantibodies, GBS is distinguished by its generally favorable prognosis and is often considered a self-limiting condition [3,4]. Most GBS patients reach their maximum disability within 2–4 weeks, followed by spontaneous neurological recovery [3,4]. As GBS and other neuroimmune disorders share similar immune-mediated damage but differ in recovery [[1], [2], [3], [4]], we used a “disease-paradigm comparison” approach to identify potential factors driving GBS recovery. Studying these factors will clarify why most neuroimmune diseases have poor prognoses and offer new insights for the development of immunotherapies.

The immune response is a protective mechanism against harmful stimuli from internal and external environments, and inflammation typically resolves once the threat has been eliminated. Over the past two decades, driven largely by the work of Serhan and colleagues, there has been growing recognition that the resolution of inflammation is an active, tightly regulated process [[5], [6], [7]]. This includes the polarization of immune cells toward anti-inflammatory phenotypes, decreased infiltration of inflammatory cells into affected tissues, and increased apoptosis of inflammatory cells. It also involves improved clearance of pathogens and cellular debris by phagocytes, along with the repair of damaged tissues [[5], [6], [7]]. The resolution of inflammation serves as the critical prerequisite and initial step for effective tissue recovery [[5], [6], [7]]. Successful resolution restores internal environment homeostasis and promotes tissue repair, whereas insufficient resolution can result in chronic inflammation and impaired regeneration [[5], [6], [7]]. Inflammation resolution is orchestrated by a class of lipid-derived molecules known as specialized pro-resolving mediators (SPMs), which are synthesized from ω-3 and ω-6 polyunsaturated fatty acids [[5], [6], [7]]. SPMs are classified into four major families: resolvins, lipoxins, protectins, and maresins. Among them, resolvin D1 (RvD1), an important member of the resolvin family, is biosynthesized from docosahexaenoic acid via enzymatic pathways catalyzed by 5-, 12-, and 15-lipoxygenases (LOXs) [8,9]. RvD1 exerts its pro-resolving effects by binding to the lipoxin A4 receptor (ALX/FPR2) and the orphan G-protein-coupled receptor 32 (GPR32), both of which are expressed on various immune cells, thereby promoting resolution and tissue repair [10].

Deficiency of RvD1 has been observed in a number of autoimmune disorders with poor prognoses [6,7]. In rheumatoid arthritis (RA), an autoimmune disease characterized by synovial inflammation [11], serum RvD1 levels are reduced [12]. Patients with systemic lupus erythematosus (SLE), a multiorgan autoimmune connective tissue disease [13], also display lower circulating levels of RvD1 than healthy controls (HCs) do [14]. These findings collectively suggest that RvD1 may serve as a biomarker for autoimmune and inflammatory diseases. The impaired resolution of inflammation caused by RvD1 deficiency may play a crucial role in the persistence of chronic inflammation or the recurrence of acute inflammatory episodes in these conditions.

Given that GBS has a more favorable prognosis than other neuroimmune disorders do, it offers an opportunity to explore factors associated with better recovery outcomes in neuroautoimmune conditions [[1], [2], [3], [4]]. The diagnostic and therapeutic potential of RvD1 in GBS remains unclear. In this study, using the GBS as a “recovery model” for neuroimmune disorders, we compared serum RvD1 levels among GBS patients, those with central nervous system (CNS) autoimmune diseases, those with non-inflammatory CNS diseases, and HCs. We also examined the correlations between RvD1 expression and clinical parameters in patients with GBS to evaluate the potential of RvD1 to serve as a biomarker for the assessment of disease severity. To assess the therapeutic potential of RvD1 in GBS and multiple sclerosis (MS), a chronic inflammatory demyelinating disease of the CNS that shares partially immune-mediated myelin sheath damage mechanisms with GBS but typically has a poorer prognosis [1], we administered RvD1 in the EAN and experimental autoimmune encephalomyelitis (EAE) models and observed its effects on clinical symptoms and autoimmune responses. With the integration of human cohort analysis and animal model investigations, this study aimed to explore the diagnostic and therapeutic roles of RvD1 in GBS. These explorations may shed light on the development of immunotherapies for GBS and broader translational strategies for neuroimmune disorders.

## Materials and methods

Details of the materials used in this study are provided in Supplementary Table 1. The key methods used in our study are described in detail in the following section, whereas other commonly used methods are described in detail in the supplementary material. These included hematoxylin and eosin (H&E) and Luxol fast blue (LFB) staining to evaluate the histopathology of mouse sciatic nerves; cytometric bead array (CBA) assays to assess cytokine concentrations in human and mouse serum; quantitative polymerase chain reaction (qPCR) to assess the expression of RvD1 synthetase and receptor in various mouse tissues; flow cytometry to evaluate splenic immune cell populations and macrophage phagocytic capacity; and western blot analysis to assess the expression of signal transduction molecules in immune cells.

### Ethics statement

For the human study, the work was carried out in accordance with The Code of Ethics of the World Medical Association. All participants provided written informed consent to donate serum samples for research purposes. For the animal experiments, all procedures were conducted in accordance with the ARRIVE guidelines and complied with the Guide for the Care and Use of Laboratory Animals issued by the National Research Council. Both the human and animal studies were approved by the Regional Ethics Committee of the First Hospital of Jilin University (Approval numbers: 2025273 for human studies and 20210590 for animal studies).

### Study population and sample collection

All enrolled participants were individuals attending the Department of Neurology, Neuroscience Centre, First Hospital of Jilin University, or individuals visiting the Health Examination Centre of the same hospital for routine health checks. Two independent cohorts were included in this study. Cohort 1, which included 128 subjects between 2015 and 2017, included 29 HCs, 21 patients with GBS, 24 with MS, 24 with neuromyelitis optica spectrum disorder (NMOSD), and 30 stroke patients. The MS and NMOSD groups presented with CNS autoimmune diseases, whereas the stroke group presented with CNS non-autoimmune diseases. Cohort 2 included 90 participants, consisting of 45 HCs and 45 GBS patients, who were recruited from 2019 to 2021. The inclusion and exclusion criteria are shown in Supplementary Table 2. Participants who met all the inclusion criteria and none of the exclusion criteria were enrolled. Data on demographics, clinical diagnosis and manifestations, laboratory and neuroimaging findings, and clinical management were obtained. Disease admission was defined as the day when the patient was hospitalized, with a strict inclusion criterion that hospitalization must have occurred within 1 week of the first clinical symptom onset. The nadir referred to the day when the patient’s GBS disability scale score reached its highest value during the disease course, and discharge was defined as the day when the patient was discharged from the hospital.

At admission, serum samples were obtained from all participants in both cohorts. For 31 of the 45 GBS patients in cohort 2, a second paired serum sample was collected 5–10 days post-admission, and this time point was designated the progression stage. Serum samples were collected following the recommended consensus protocol for serum collection and biobanking [15]. In brief, peripheral venous blood was collected in a vacuum tube containing a coagulant and then centrifuged at 2000 × g for 10 min at 4 °C. No more than 4 h of temporal storage at 4 °C was permitted between blood collection and centrifugation. The serum was carefully collected, aliquoted into 0.5 mL polypropylene tubes, and stored at −80 °C until further analysis.

### Assessment of disease severity in GBS patients

The clinical severity and functional impairment of GBS patients were assessed via two widely used scales: the GBS disability scale (Supplementary Table 3) [16] and the Medical Research Council (MRC) sum score [17]. Limb weakness was assessed via the MRC sum score, which evaluates six bilateral muscle groups in the upper and lower limbs, with a total score ranging from 0 (tetraplegia) to 60 (normal strength) [17].

### Disease model induction, severity evaluation and drug administration

All the mice were housed under barrier conditions with a controlled temperature (22 ± 2 °C), relative humidity (50–60 %), and a 12-hour light/dark cycle, with food and water available ad libitum. Consistent with common practices and human epidemiology, the EAN model was induced in male C57BL/6J mice aged 8–10 weeks and weighing 18–20 g, whereas the EAE model was induced in age- and weight-matched female mice. EAN mice were subcutaneously immunized with 200 μg of P0 peptide (residues 180–199), and EAE mice were immunized with 200 μg of myelin oligodendrocyte glycoprotein peptide 35–55 (MOG_35–55_), both of which were emulsified in 200 μl of complete Freund's adjuvant supplemented with heat-inactivated *Mycobacterium tuberculosis* (5 mg/ml for EAN; 4 mg/ml for EAE). Pertussis toxin was administered intraperitoneally at the time of immunization and again 48 h later: 400 ng for EAN and 300 ng for EAE. Clinical severity was scored daily by two investigators who were blinded to the treatment groups (Supplementary Table 3).

For treatment, EAN mice were randomly assigned to the “RvD1” or “EAN” group. The mice in the RvD1 group received 5 μg/kg RvD1 in phosphate-buffered saline (PBS) via intraperitoneal injection for five consecutive days starting from day 8 post-immunization, whereas those in the EAN group received an equal volume of PBS alone. Similarly, EAE mice were allocated to the “RvD1” or “EAE” group and received daily intraperitoneal injections of either 5 μg/kg RvD1 in PBS or PBS alone from day 8 to day 17 post-immunization. The RvD1 dose was determined on the basis of previously published studies [12,18]. Age- and sex-matched healthy mice were used as the “Control” group and received PBS only.

### Enzyme-linked immunosorbent assay

The mice were sacrificed at different time points during the disease course. Spleens and bilateral sciatic nerves were harvested, blotted dry with filter paper, weighed, and snap-frozen in liquid nitrogen. The tissues were ground to powder and homogenized in PBS (1:5 w/v). The homogenates were then sonicated and centrifuged at 12,000 × g for 10 min, and the supernatants were collected for analysis.

The concentrations of the anti-inflammatory lipid mediators RvD1 and lipoxin A4 and the pro-inflammatory mediator leukotriene B4 were measured in human serum, mouse serum, the spleen, and sciatic nerves via acetylcholinesterase (AChE)-based competitive enzyme-linked immunosorbent assay (ELISA). Briefly, samples and standards were incubated in a mouse anti-rabbit IgG precoated 96-well plate along with the lipid–AChE conjugates and antibody. After washing, Ellman’s reagent was added for development. The absorbance was measured at 414 nm via a microplate reader (BIO-TEK, USA), and the concentrations were calculated from standard curves. The anti-inflammatory protein mediator annexin A1, which shares a receptor with RvD1, was assessed via sandwich ELISA according to the manufacturer’s instructions.

### Statistical analysis

All the quantitative data were first tested for normality using the Shapiro–Wilk test. Normally distributed data are expressed as the mean ± standard error of the mean (SEM). For paired comparisons, the paired *t* test was used; for comparisons between two independent groups, the independent-samples *t* test was applied. For comparisons among three or more groups, one-way analysis of variance (ANOVA) followed by the least significant difference (LSD) post hoc test was conducted. Correlations between two variables were analyzed via Pearson’s correlation coefficient. For non-normally distributed or qualitative data, the results are expressed as medians with interquartile ranges (IQRs). Comparisons between two groups were performed using the Mann–Whitney *U* test, and comparisons among three or more groups were analyzed via the Kruskal–Wallis H test. When the Kruskal–Wallis test indicated significant differences, pairwise comparisons were performed using the Mann–Whitney *U* test with Bonferroni correction. Correlation analysis for non-parametric data was conducted using Spearman’s rank correlation coefficient. Differences in proportions were analyzed via the chi-square test. Receiver operating characteristic (ROC) curve analysis was used to evaluate the performance of binary classification models by illustrating the trade-off between the true positive rate (sensitivity) and false positive rate (1-specificity) across different decision thresholds. Multiple linear regression was used to analyze the associations between the continuous dependent variable and multiple independent variables. Post hoc power analysis was performed to assess the adequacy of sample sizes for group comparisons. The practical effect size was assessed via Cohen’s d, with the following classification of effect magnitudes: Cohen’s d ≤ 0.2 indicates a small effect; 0.2 < Cohen’s d < 0.8 indicates a moderate effect; and Cohen’s d ≥ 0.8 indicates a large effect.

The post hoc power analysis was conducted via PASS version 15.0 (NCSS, Kaysville, UT, USA), whereas other statistical analyses were performed via SPSS version 28.0 (IBM Deutschland, Ehningen, Germany), and figures were created using GraphPad Prism version 8.0 (GraphPad Software Inc., San Diego, CA, USA). All tests were two-tailed, and *p* values ≤ 0.05 were considered statistically significant.

## Results

### Serum RvD1 is elevated in patients with GBS compared with HCs and those with other CNS diseases

The demographic characteristics of the enrolled subjects, including sex and age distribution, are summarized in Table 1. In cohort 1, a total of 21 patients with GBS, 29 HCs, 30 stroke patients, 24 NMOSD patients, and 24 MS patients were enrolled. Patients in the GBS group were older and more likely to be male than those in the MS and NMOSD groups were (Table 1). In cohort 2, 45 GBS patients and 45 age- and sex-matched HCs were recruited, with no significant demographic differences between the groups (Table 1).

**Table 1:** Demographic characteristics of enrolled subjects.

**Cohorts**	**Sex, number (male/female)**	***P* value** **(vs GBS)**	**Age, years** **(median with IQR)**	***P* value** **(vs GBS)**
**Cohort 1**				
HC	29 (18/11)	0.738	47 (41.5–64.5)	0.768
GBS	21 (14/7)	N/A	56 (46.5–62.5)	N/A
Stroke	30 (20/10)	1	59 (51–67.5)	0.25
NMOSD	24 (8/16)	0.026*	49.5 (30.75–53)	0.012*
MS	24 (5/19)	0.002**	37.5 (30.25–44.5)	< 0.001***
**Cohort 2**				
HC	45 (24/21)	0.523	55 (41.5–69)	0.786
GBS	45 (27/18)	N/A	54 (38–69.5)	N/A

GBS = Guillain–Barré syndrome; HC = healthy control; IQR = interquartile range; MS = multiple sclerosis; NMOSD = neuromyelitis optica spectrum disorders; N/A = Not available. *p ≤ 0.05; **p < 0.01; ***p < 0.001.

**Fig. 1:**
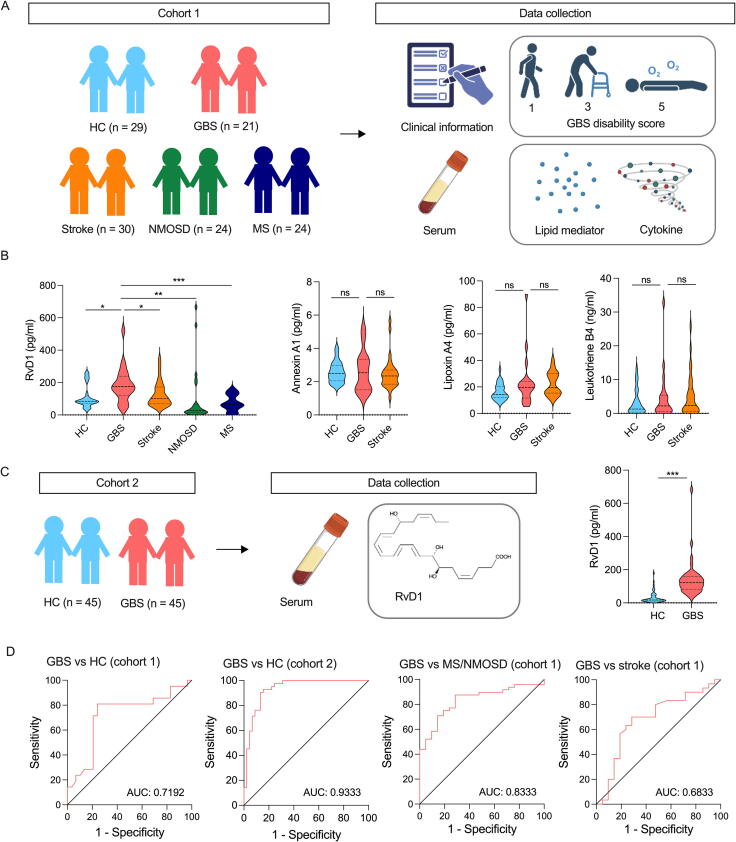
**Serum levels of RvD1 are elevated in patients with GBS.** A total of 128 participants were enrolled in cohort 1, comprising 29 HCs, 21 patients with GBS, 30 stroke patients, 24 patients with NMOSD, and 24 with MS. Demographic information for all the subjects and clinical data for the GBS patients were collected. Serum levels of lipid mediators and cytokines were quantified. The figure was created via BioRender (https://biorender.com). (B) Serum concentrations of RvD1 were significantly greater in the GBS patients than in the HCs, stroke patients, NMOSD patients, and MS patients. No significant differences were observed in the levels of annexin A1, lipoxin A4, or leukotriene B4 among the HC, GBS, and stroke groups. (C) In cohort 2, 45 HCs and 45 GBS patients were recruited. The RvD1 levels were significantly elevated in the GBS group. (D) ROC curves showing the high sensitivity and specificity of RvD1 in distinguishing GBS patients from HCs, MS/NMOSD patients, and stroke patients. Comparisons of RvD1 levels among different diagnostic groups in cohort 1 were performed via one-way ANOVA followed by LSD post hoc tests. Comparisons between the GBS and HC groups in cohort 2 were performed via independent-samples t tests. ROC curve analysis was used to evaluate the diagnostic performance, illustrating the trade-off between sensitivity (true positive rate) and 1-specificity (false positive rate). ANOVA = analysis of variance; AUC = area under the curve; GBS = Guillain–Barré syndrome; HC = healthy control; MS = multiple sclerosis; NMOSD = neuromyelitis optica spectrum disorders; ns = not significant; LSD = least significant difference; RvD1 = resolvin D1; ROC = receiver operating characteristic. *p ≤ 0.05; **p < 0.01; ***p < 0.001.

We first compared the serum concentrations of RvD1, annexin A1, lipoxin A4, and leukotriene B4 among the GBS, HC, and stroke groups in cohort 1. RvD1 levels were significantly elevated in GBS (mean 185.33 pg/ml) patients compared with both HCs (mean 110.23 pg/ml, *p* = 0.011) and stroke patients (mean 127.70 pg/ml, *p* = 0.048) (Fig. 1, Fig. 1). Next, we compared the serum RvD1 levels in patients with GBS with those in patients in the NMOSD and MS groups. The RvD1 levels were significantly higher in the GBS group than in both the NMOSD (mean 98.99 pg/ml, *p* = 0.005) and MS (mean 74.94 pg/ml, *p* < 0.001) groups (Fig. 1B). To assess whether sex or age influenced the observed differences in RvD1 levels, we performed multiple linear regression analysis, which revealed that RvD1 levels were not significantly associated with sex or age. Consistently, the comparison of RvD1 levels between males and females in cohort 1 revealed no significant differences, and the correlation analysis also revealed no significant association with age. These results indicated that sex and age were not confounding factors in the RvD1 analysis. We then validated the findings in cohort 2. Consistent with those in cohort 1, RvD1 concentrations were significantly higher in GBS patients (mean 139.16 pg/ml) at admission than in HCs (mean 32.72 pg/ml, *p* < 0.001) (Fig. 1C). Post hoc power analysis was performed to evaluate whether the current sample size was sufficient for comparing RvD1 levels between the GBS and non-GBS groups (HC, stroke, MS, and NMOSD patients pooled as non-GBS patients) in cohort 1 and between the GBS and HC groups in cohort 2. The statistical power reached 100 % in both cohorts, indicating adequate sample sizes.

We subsequently investigated the sensitivity and specificity of RvD1 in differentiating GBS patients from HCs, patients with CNS autoimmune diseases, and patients with CNS non-autoimmune diseases. ROC curve analysis demonstrated that RvD1 exhibited high sensitivity and specificity for distinguishing GBS patients from HCs (area under the curve [AUC] = 0.7192 in cohort 1; AUC = 0.9333 in cohort 2), MS patients and NMOSD patients (AUC = 0.8333 in cohort 1), and stroke patients (AUC = 0.6833 in cohort 1) (Fig. 1D). In summary, serum RvD1 levels are significantly elevated in patients with GBS and have high sensitivity and specificity in differentiating patients with GBS from HCs, those with CNS autoimmune diseases, and those with CNS non-autoimmune diseases. The optimal cutoffs with 95 % confidence intervals (CIs) are presented in Supplementary Table 4. These findings suggest that RvD1 has the potential to serve as a diagnostic biomarker for GBS.

### The RvD1 resolution axis is upregulated during the disease course of GBS and EAN

#### RvD1 levels increase as GBS progresses

**Fig. 2:**
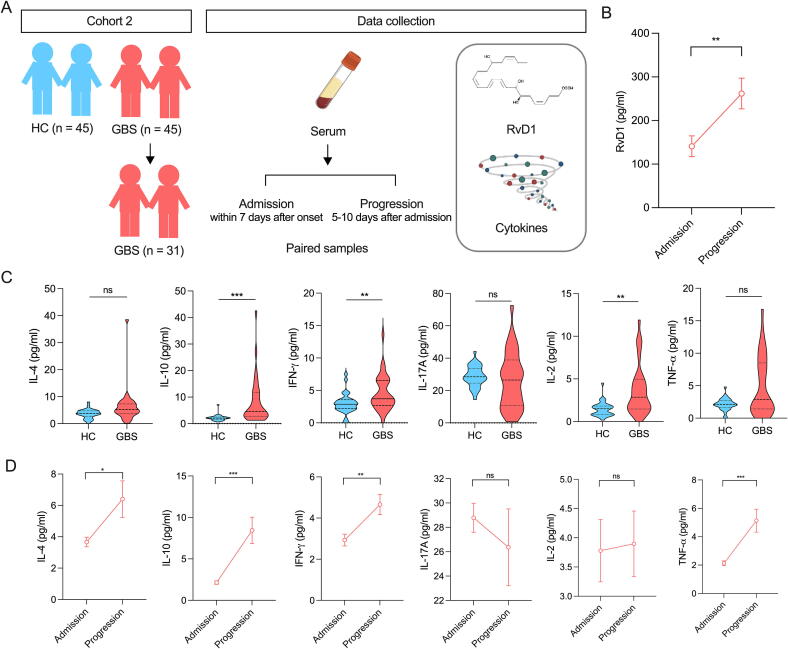
**The serum RvD1 level in patients with GBS increases as the disease progresses.** (A) A total of 45 HCs and 45 GBS patients were enrolled in cohort 2. Among these 45 GBS patients, 31 had paired serum samples collected at both the admission and progression stages. Disease admission was defined as the day when the patient was hospitalized, with a strict inclusion criterion that hospitalization must have occurred within 1 week of the first clinical symptom onset. The progression stage was defined as 5–10 days after admission. The levels of RvD1 and cytokines were assessed at both admission and progression. The figure was created via BioRender (https://biorender.com). (B) Serum RvD1 levels were significantly elevated during the progression stage compared with those at admission. (C) Serum concentrations of IL-10, IFN-γ, and IL-2 were significantly greater in GBS patients than in HCs. (D) The levels of anti-inflammatory cytokines (IL-4 and IL-10) and pro-inflammatory cytokines (IFN-γ and TNF-α) increased as GBS progressed. Paired t tests were applied for comparisons within the same individuals (admission vs. progression), and independent-samples t tests were used for comparisons between the GBS and HC groups. GBS = Guillain–Barré syndrome; HC = healthy control; IFN = interferon; IL = interleukin; ns = not significant; RvD1 = resolvin D1; TNF = tumor necrosis factor. *p ≤ 0.05; **p < 0.01; ***p < 0.001.

**Supplementary Figure S1:**
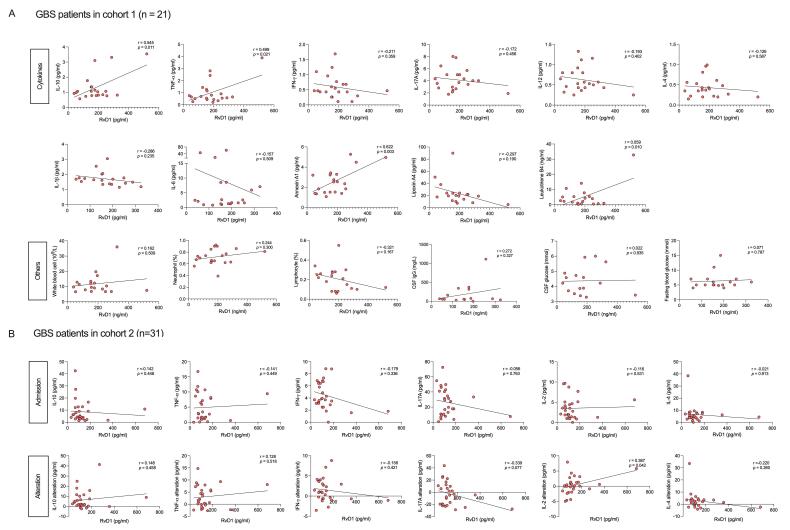
Correlations between RvD1 levels and other immune mediators. (A) Correlation analysis of RvD1 levels with cytokines and immune parameters in cohort 1. (B) Correlation analysis of RvD1 levels with cytokines measured at admission and with cytokine changes from admission to progression in cohort 2. Correlations between two variables were analyzed via Pearson’s correlation coefficient. CSF = cerebrospinal fluid; IFN = interferon; IL = interleukin; RvD1 = resolvin D1; TNF = tumor necrosis factor.

Among the 45 patients with GBS in cohort 2, 31 had paired serum samples collected at admission (within 7 days after onset) and during the progressive phase (5–10 days after admission) (Fig. 2A). We observed a significant increase in the serum RvD1 level as GBS progressed (mean 141.00 pg/ml vs*.* 247.65 pg/ml, *p* = 0.009) (Fig. 2B). We further analyzed cytokine alterations during disease progression. Compared with those in HCs, the levels of interleukin (IL)-10, interferon (IFN)-γ, and IL-2 were elevated at admission in patients with GBS (Fig. 2C). Both anti-inflammatory cytokines (IL-4, IL-10) and pro-inflammatory cytokines [IFN-γ, tumor necrosis factor (TNF)-α] were significantly elevated as the disease progressed (Fig. 2D and Supplementary Table 5). We assessed the correlations between RvD1 and cytokines or other lipid mediators. In cohort 1, RvD1 levels were positively correlated with IL-10, TNF-α, annexin A1, and leukotriene B4 concentrations (Supplementary Fig. 1A). In contrast, in cohort 2, RvD1 levels at admission were not correlated with any cytokines measured at the same time point (Supplementary Fig. 1B). We then examined whether the RvD1 level at admission was associated with cytokine changes from admission to progression and observed a positive correlation with IL-2 alterations (Supplementary Fig. 1B).

#### Upregulation of the RvD1 resolution axis in EAN

**Supplementary Figure S2:**
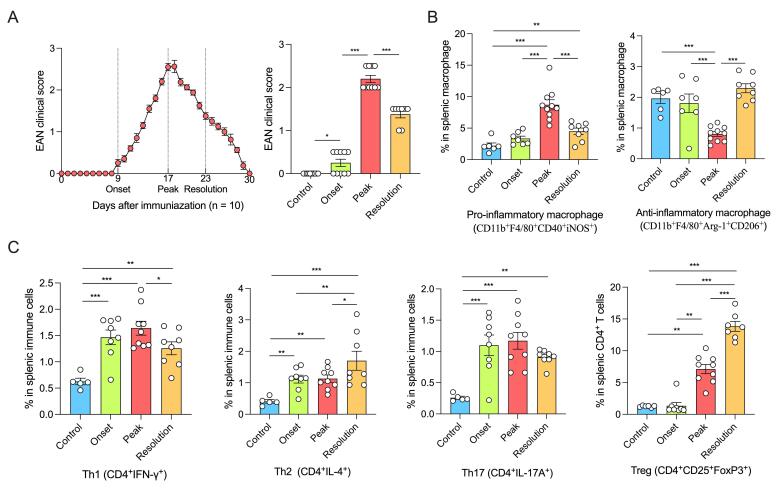
Disease course of EAN. (A) The EAN clinical score started to increase at onset, reached maximum at peak, and decreased at resolution stage. (B) The proportion of pro-inflammatory macrophages in the spleen increased at the disease peak and declined during resolution. In the contrast, the corresponding proportion of anti-inflammatory macrophage decreased at disease peak, then started to increase at resolution phase. (C) The percentages of Th1, Th2 and Th17 cells in splenic T cells were elevated at all disease stages compared to control. Treg cells were markedly increased at peak and resolution stages. Comparisons among three or more groups were performed via one-way ANOVA followed by LSD post hoc tests. ANOVA = analysis of variance; Arg-1 = arginase-1; EAN = experimental autoimmune neuritis; FoxP3 = Forkhead box protein P3; IFN = interferon; IL = interleukin; iNOS = inducible nitric oxide synthase; LSD = least significant difference; RvD1 = resolvin D1; Th = T helper; Treg = regulatory T cell. *p ≤ 0.05; **p < 0.01; ***p < 0.001.

We employed EAN to investigate the temporal dynamics of RvD1, along with its synthetase 12/15-LOX and receptor ALX/FPR2, during the disease course. Clinical symptoms of EAN begin to manifest around day 9 post-induction (defined as disease onset), peak around day 17 (defined as the peak of disease), and significantly decline by day 23 (defined as the resolution phase) (Supplementary Fig. 2A). Supplementary Fig. 2B and C show the polarization of macrophages and T cells throughout the disease course.

**Fig. 3:**
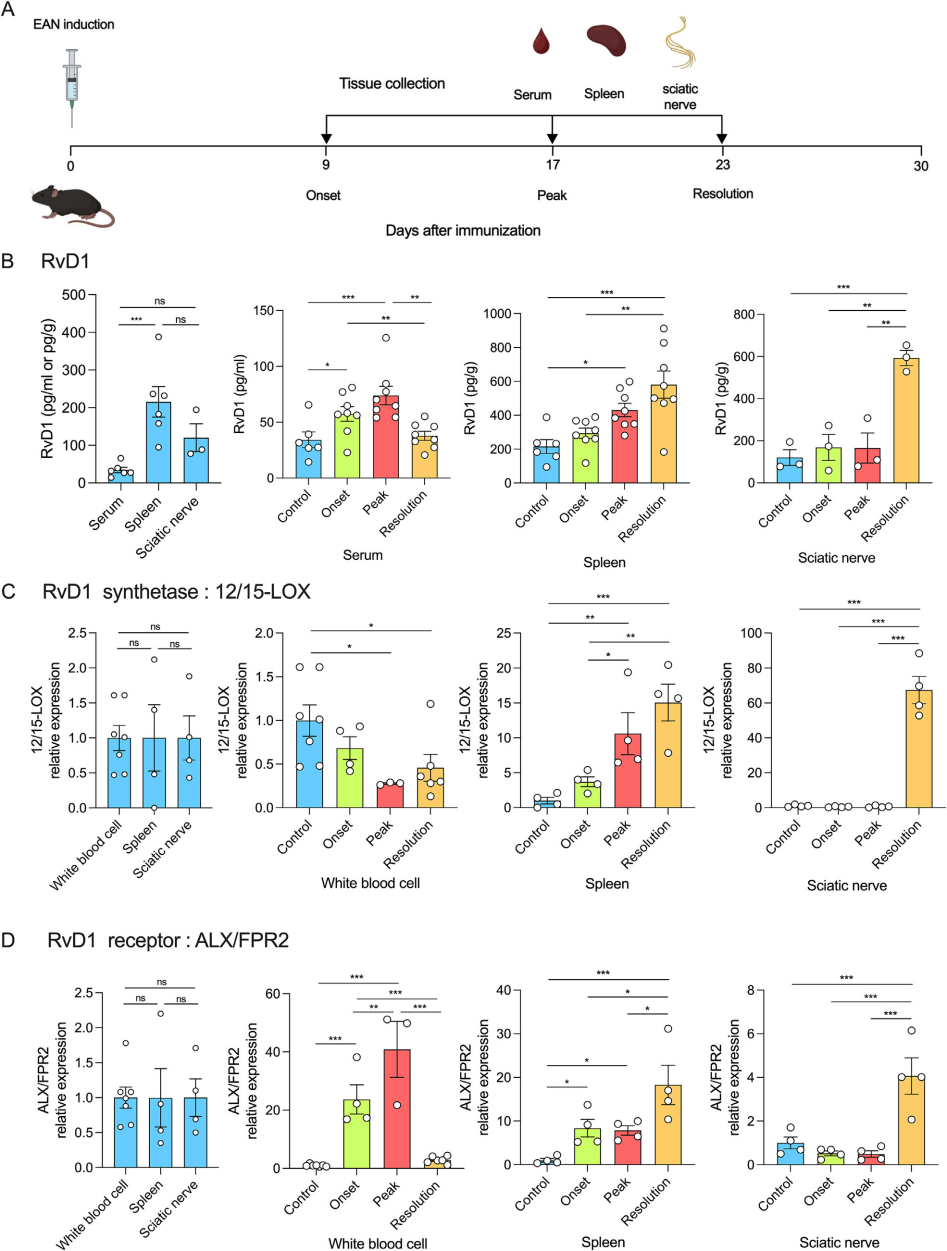
**Dynamic changes in RvD1 and its synthetase/receptor during EAN.** (A) To investigate the dynamic changes in the RvD1 resolution axis, EAN mice were sacrificed at three disease stages: onset (9 days post-immunization), peak (17 days post-immunization), and resolution (23 days post-immunization). Serum, spleen, white blood cells, and sciatic nerve samples were collected for RvD1 quantification and analysis of its biosynthetic enzyme 12/15-LOX and receptor ALX/FPR2. The figure was created via BioRender (https://biorender.com). (B) The expression levels of RvD1 in healthy mice were higher in the spleen than in the serum. In the serum, the RvD1 level increased after onset, reached a maximum at peak, and then started to decrease in the resolution phase. In the spleen, RvD1 levels continued to rise at onset and peak and reached a maximum in the resolution phase. In the sciatic nerves, the levels of RvD1 remained unchanged at disease onset and peak, whereas they significantly increased in the resolution stage. (C) The expression of the RvD1 synthetase 12-/15-LOX was distinct across white blood cells, the spleen, or the sciatic nerves in control mice. During EAN, 12-/15-LOX expression decreased at the peak and resolution phases in white blood cells, increased in the spleen at the peak and resolution stages, and was upregulated in sciatic nerves during resolution. (D) Baseline expression of the RvD1 receptor ALX/FPR2 was comparable among control mouse tissues. During EAN, ALX/FPR2 expression increased at onset and peak in white blood cells, followed by a decrease in resolution. In the spleen, expression rose consistently across all stages, whereas in sciatic nerves, upregulation was observed only during resolution. One-way ANOVA followed by the LSD post hoc test was used for comparisons among three or more groups. ALX/FPR2 = lipoxin A4 receptor; ANOVA = analysis of variance; EAN = experimental autoimmune neuritis; LOX = lipoxygenase; ns = not significant; LSD = least significant difference; RvD1 = resolvin D1. *p ≤ 0.05; **p < 0.01; ***p < 0.001.

Serum, spleen, white blood cells, and sciatic nerves were collected from EAN mice at the onset, peak, and resolution stages (Fig. 3A). In the serum, RvD1 levels were significantly elevated at the peak of the disease, which is consistent with findings in patients with GBS (Fig. 3B). In white blood cells, the level of RvD1 synthetase was significantly decreased, while that of its receptor was increased at the peak of disease, indicating that white blood cells may function primarily as RvD1 responders during this stage (Fig. 3C and D). The entire RvD1 resolution axis was upregulated in the spleen at both the peak and resolution stages, whereas in the sciatic nerve, it was elevated only at the resolution stage (Fig. 3C and D). These findings suggest that the RvD1 resolution axis may limit systemic inflammation during the progression phase while promoting tissue regeneration within the peripheral nervous system in the resolution phase.

### RvD1 levels are associated with GBS severity, ventilator dependence and axonal neuropathy

**Fig. 4:**
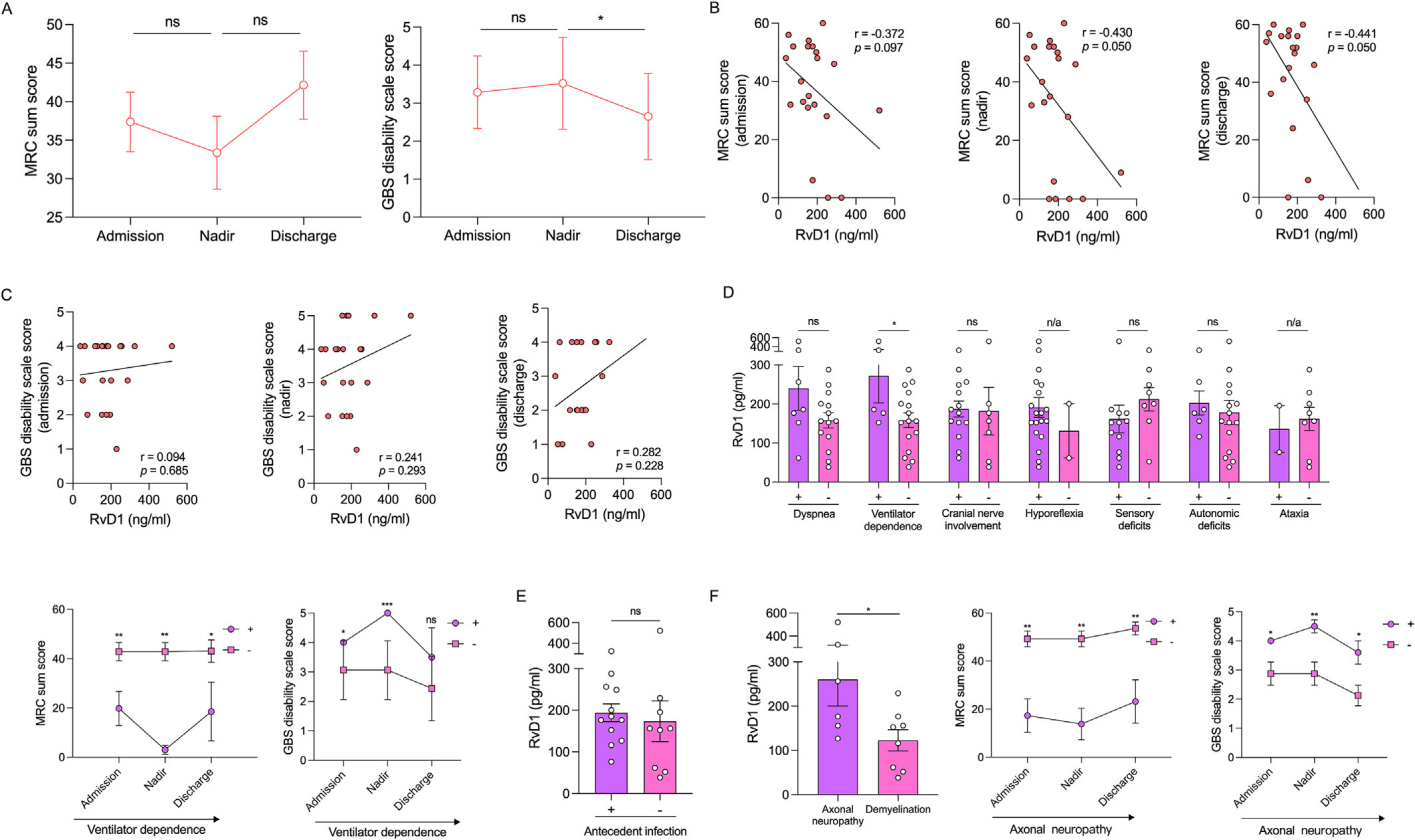
**RvD1 levels are associated with GBS severity, ventilator dependence and axonal neuropathy.** Using data from cohort 1, the associations between serum RvD1 levels at admission and the clinical parameters of patients with GBS were analyzed. (A) GBS severity was evaluated by the MRC sum score and the GBS disability scale. The MRC score declined at nadir and improved at discharge. The disability scale score significantly improved from nadir to discharge. (B) MRC scores at nadir and discharge were negatively correlated with serum RvD1 levels. (C) No significant correlation was detected between RvD1 levels and GBS disability scale scores. (D) RvD1 levels were significantly elevated in GBS patients who required ventilator support. Ventilator-dependent GBS patients had a more severe disease course. (E) RvD1 concentrations did not differ between patients with and without antecedent infections. (F) GBS patients with axonal neuropathy, who typically present with more severe disease, had significantly higher RvD1 levels than patients with the demyelinating subtype did. Mann‒Whitney U tests were used to compare disease severity between admission and nadir, as well as between nadir and discharge. Independent-samples t tests were used for comparisons between different GBS subgroups. Correlation analyses for non-parametric data were conducted via Spearman’s rank correlation coefficient. MRC = Medical Research Council; ns = not significant; RvD1 = resolvin D1. *p ≤ 0.05; **p < 0.01; ***p < 0.001.

We investigated the associations between serum RvD1 levels at admission and clinical parameters in patients with GBS using data from cohort 1. Disease severity at admission, nadir, and discharge was assessed via the MRC sum score and GBS disability scale (Fig. 4A). Serum RvD1 levels were negatively correlated with MRC sum scores at nadir and at discharge, suggesting that a more severe disease state provoked more RvD1 secretion (Fig. 4B and C). We further analyzed the relationship between RvD1 and the clinical manifestations of GBS (Table 2). Patients who required ventilator support—a marker of more severe disease—had significantly higher serum RvD1 levels (Cohen’s d = 1.159) (Fig. 4D). RvD1 levels were not significantly different between patients with or without cranial nerve involvement, sensory deficits, autonomic dysfunction or antecedent infections (Fig. 4D and E). We analyzed RvD1 levels across GBS subtypes, which were classified as either the axonal neuropathy (including acute motor axonal neuropathy (AMAN) and acute motor and sensory axonal neuropathy (AMSAN)) or the demyelinating subtype (acute inflammatory demyelinating polyneuropathy (AIDP)). Patients with axonal neuropathy presented significantly elevated RvD1 levels and more severe clinical symptoms than patients with the demyelinating subtype did (Cohen’s d = 1.273) (Fig. 4F). Owing to limited clinical data, these analyses were not conducted in cohort 2.

**Table 2:** Clinical information of the GBS patients in cohort 1.

**Clinical manifestations**	**Percentage** **(involved/total patients number)**
Dyspnea	33.33 % (7/21)
Ventilator dependence	23.81 % (5/21)
Cranial nerve involvement	66.67 % (14/21)
Hyporeflexia	90.48 % (19/21)
Sensory deficits	57.14 % (12/21)
Autonomic deficits	28.57 % (6/21)
Ataxia	10.00 % (2/20)
**Symptoms of antecedent infection**	57.14 % (12/21)
**CSF examinations**	
Mean protein concentration (g/L)	1.53
Albumin-cytologic dissociations	93.75 % (15/16)
Mean IgG concentration (mg/L)	183.97
**Nerve conduction studies**	
Demyelination (AIDP)	57.14 % (8/14)
Axonal neuropathy (AMAN or AMSAN)	42.86 % (6/14)

AIDP = acute inflammatory demyelinating polyneuropathy; AMAN = acute motor axonal neuropathy; AMSAN = acute motor–sensory axonal neuropathy; CSF = cerebrospinal fluid; GBS = Guillain–Barré syndrome; IgG = immunoglobulin G.

In summary, elevated serum RvD1 levels are associated with increased disease severity, ventilator dependence, and axonal pathology in patients with GBS. These findings suggest the potential of RvD1 as a biomarker for disease severity.

### Administration of RvD1 ameliorates EAN and EAE

**Fig. 5:**
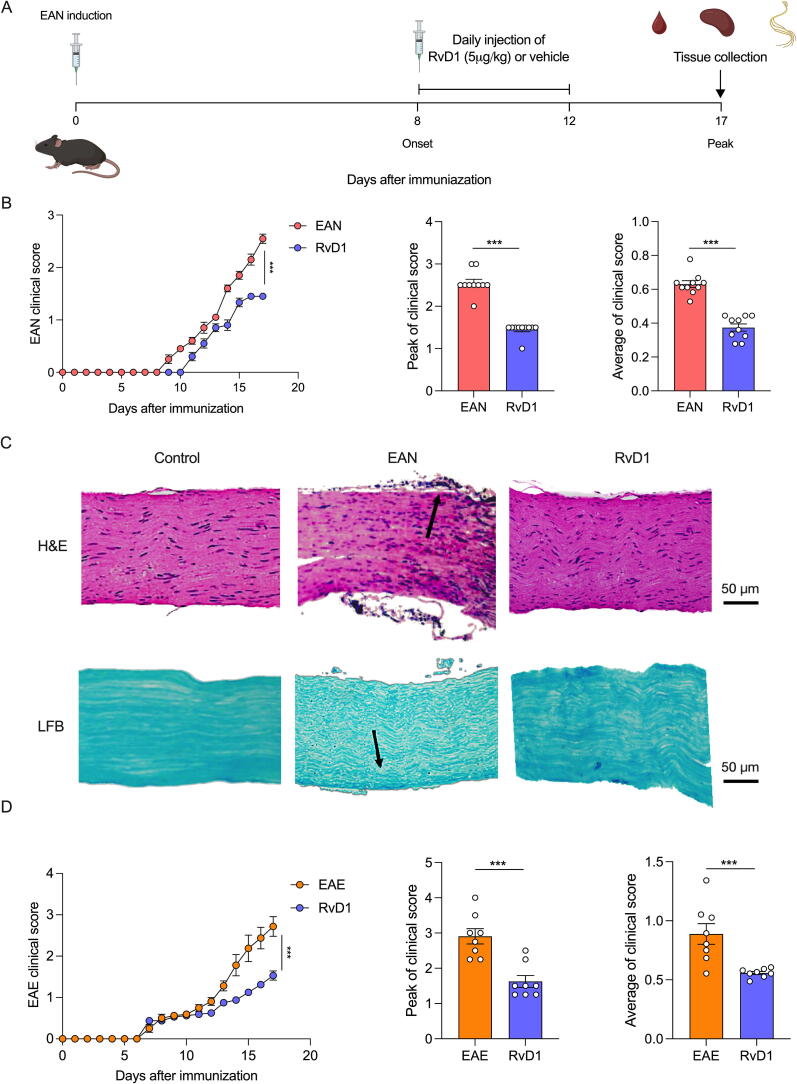
**Administration of RvD1 alleviates disease severity and histopathology in EAN.** (A) EAN mice received daily intraperitoneal injections of RvD1 or vehicle from days 9 to 13 post-immunization. Samples were collected from the blood, spleen, and sciatic nerves on day 17. The figure was created via BioRender (https://biorender.com). (B) Compared with vehicle-treated control mice, RvD1-treated mice presented a milder disease course. (C) Histological analysis revealed reduced immune cell infiltration and demyelination in the sciatic nerves of RvD1-treated mice. (D) Compared with untreated EAE mice, RvD1-treated mice presented a significantly milder disease course. Clinical score comparisons between two groups were performed via the Mann‒Whitney *U* test. EAE = experimental autoimmune encephalomyelitis; EAN = experimental autoimmune neuritis; H&E = hematoxylin and eosin; LFB = Luxol fast blue; ns = not significant; RvD1 = resolvin D1. ***p < 0.001.

To assess the therapeutic efficacy of RvD1 in EAN, clinical signs were monitored daily in both RvD1- and vehicle-treated mice (Fig. 5A). Compared with vehicle treatment, RvD1 treatment significantly alleviated clinical symptoms, as indicated by reduced maximum and average clinical scores (Fig. 5B). To determine whether the clinical improvement was accompanied by reduced histopathological changes, we performed H&E and LFB staining to assess inflammatory cell infiltration and demyelination in the sciatic nerves. Compared with vehicle treatment, RvD1 treatment markedly reduced inflammatory cell infiltration and myelin loss in EAN model mice (Fig. 5C). Collectively, these findings demonstrate that RvD1 mitigated both disease severity and nerve pathology in EAN.

To assess whether the therapeutic effect of RvD1 extends to other autoimmune conditions of the CNS, we administered RvD1 in EAE and compared disease severity between RvD1- and vehicle-treated mice (Fig. 5D). Compared with vehicle-treated control EAE mice, RvD1-treated EAE mice presented significantly lower average and peak clinical scores (Fig. 5D).

### Treatment with RvD1 promotes the resolution of inflammation in EAN

**Fig. 6:**
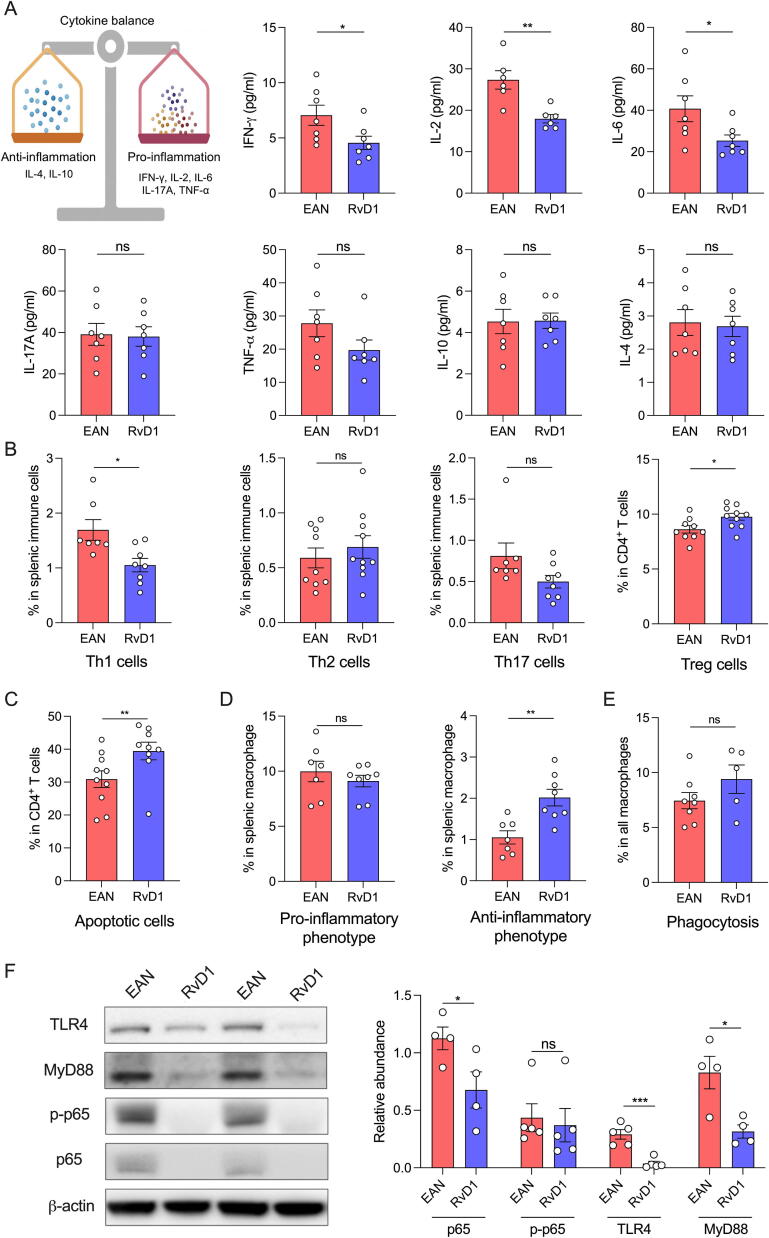
**Treatment with RvD1 attenuates autoimmune responses in EAN.** (A) The levels of the anti-inflammatory cytokines IL-4 and IL-10 and the pro-inflammatory cytokines IFN-γ, IL-2, IL-6, IL-17A, and TNF-α were quantified in mouse serum. RvD1 treatment significantly suppressed the levels of IFN-γ, IL-2, and IL-6. (B) RvD1 administration decreased the Th1 cell frequency and increased the Treg proportion. (C) RvD1 enhanced the apoptosis of CD4^+^ T cells in the spleen. (D) RvD1 increased the proportion of anti-inflammatory macrophages in the spleen. (E) Macrophage phagocytic capacity was not affected by RvD1 treatment. (F) Macrophages from RvD1-treated EAN mice expressed reduced levels of p65, TLR4, and MyD88. Comparisons between the EAN and RvD1 groups were performed via independent-samples t tests. EAN = experimental autoimmune neuritis; IFN = interferon; IL = interleukin; ns = not significant; RvD1 = resolvin D1; Th = T helper; TLR = Toll-like receptor; TNF = tumor necrosis factor; Treg = regulatory T cell. *p ≤ 0.05; **p < 0.01; ***p < 0.001.

**Supplementary Figure S3:**
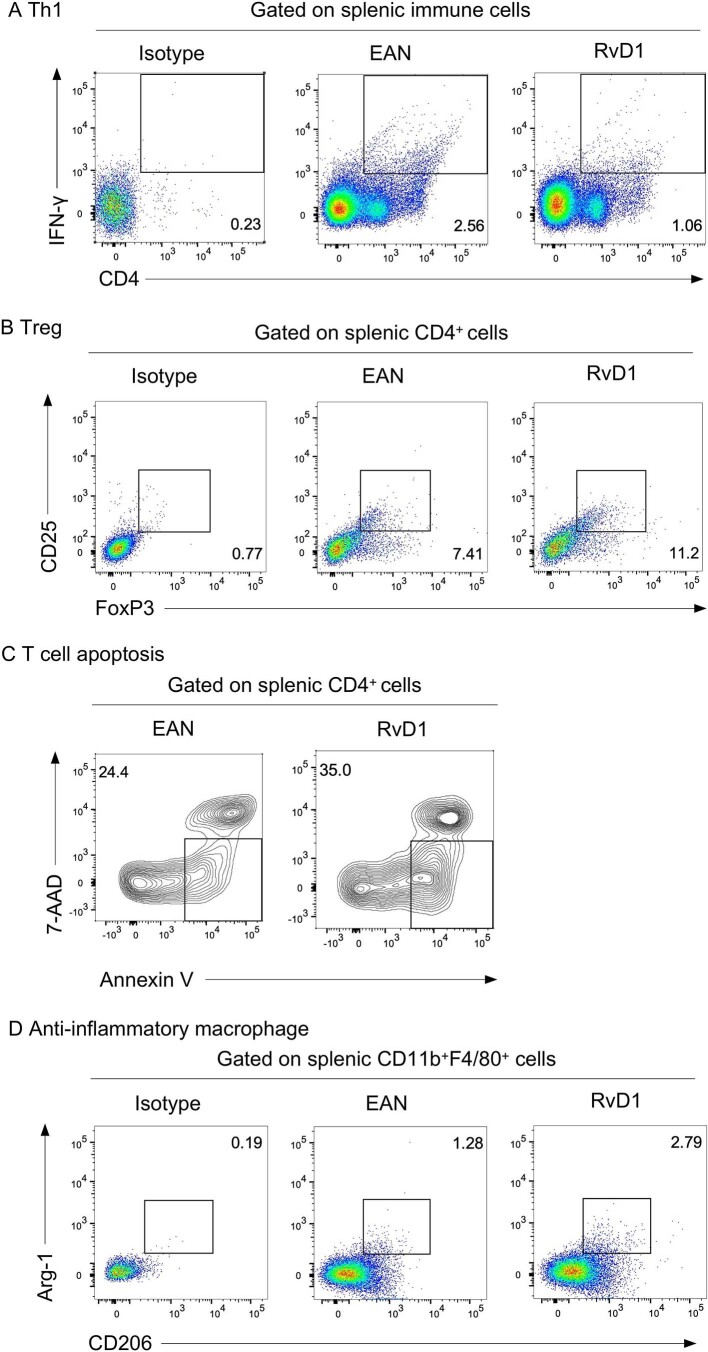
Gating strategies for flow cytometry. Splenic immune cells were isolated from EAN mice treated with either vehicle (EAN group) or RvD1 (RvD1 group) for flow cytometry analysis. (A) Gating strategy for Th1 cells (CD4^+^IFNγ^+^) (n = 7, EAN group; n = 8, RvD1 group). (B) Gating strategy for Treg cells (CD4^+^CD25^+^FoxP3^+^) (n = 9, EAN group; n = 10, RvD1 group). (C) Gating strategy for early apoptotic T cells (7-AAD^-^Annexin V^+^) (n = 10, EAN group; n = 9, RvD1 group). (D) Gating strategy for anti-inflammatory macrophages (CD11b^+^F4/80^+^Arg^+^CD206^+^) (n = 7, EAN group; n = 8, RvD1 group). 7-AAD = 7-Aminoactinomycin D; Arg-1 = arginase-1; EAN = experimental autoimmune neuritis; FoxP3 = Forkhead box protein P3; IFN = interferon; RvD1 = resolvin D1; Th = T helper; Treg = regulatory T cell.

To evaluate whether RvD1 reduces overall inflammatory responses, serum cytokine levels were measured at the peak of the disease. RvD1 treatment significantly decreased the levels of IFN-γ, IL-2, and IL-6 (Fig. 6A). To explore the effects of RvD1 on T-cell polarization and apoptosis, we performed flow cytometric analysis of T-cell subsets in the spleen. Compared with vehicle treatment, RvD1 treatment significantly reduced the proportion of pro-inflammatory T helper (Th) 1 cells while increasing the percentage of anti-inflammatory regulatory T (Treg) cells (Fig. 6B and Supplementary Fig. 3A-B). The proportion of early apoptotic CD4^+^ T cells was significantly greater in the RvD1-treated group than in the control group (Fig. 6C and Supplementary Fig. 3C).

To assess the impact of RvD1 on macrophage polarization and phagocytosis, flow cytometry was used to analyze the phenotype and function of macrophages from the spleen and peritoneal cavity, respectively. We found that RvD1 treatment increased the proportion of anti-inflammatory macrophages (Fig. 6D and Supplementary Fig. 3D). No significant difference in phagocytic capacity was observed between the two groups (Fig. 6E). To investigate the mechanisms underlying the pro-resolving effects of RvD1 on macrophages, we measured the expression of key signaling molecules via western blotting. RvD1 treatment significantly downregulated the expression of Toll-like receptor 4 (TLR4), MyD88, and total p65 in macrophages (Fig. 6F).

In summary, RvD1 promoted the resolution of inflammation in EAN by reducing pro-inflammatory cytokine expression in the serum, facilitating anti-inflammatory T-cell polarization, increasing T-cell apoptosis, and shifting macrophages toward an anti-inflammatory phenotype without altering their phagocytic capacity.

## Discussion

Unlike most neuroimmune diseases, which typically follow a progressive or relapsing–remitting course, GBS generally has a favorable prognosis. However, the mechanisms underlying the spontaneous recovery observed in patients with GBS remain largely elusive. In this study, we found that serum levels of RvD1 were elevated in patients with GBS and exhibited good diagnostic accuracy in distinguishing patients with GBS from HCs, those with CNS autoimmune diseases and those with non-autoimmune CNS diseases. Furthermore, the RvD1 level in the serum continued to increase as GBS progressed; this pattern was further supported by findings in EAN, where the serum RvD1 level was elevated at onset and rose further at the peak stage. Serum RvD1 levels were positively associated with disease severity, ventilator dependence, and the presence of axonal neuropathy. Administration of RvD1 significantly ameliorated both EAN and EAE. Collectively, these findings suggest that RvD1 may serve as both a diagnostic biomarker and therapeutic agent for GBS and EAN. Owing to its capacity for spontaneous recovery, GBS may serve as a model for exploring resolution mechanisms in autoimmune diseases. Elucidating molecules associated with recovery in patients with GBS may facilitate the identification of novel therapeutic strategies.

In contrast to their reduction or lack of change in other pathological conditions, RvD1 levels are elevated in patients with GBS, highlighting its potential utility as a diagnostic marker. A previous study revealed no significant change in the serum RvD1 level in MS patients compared with HCs [19]. In RA and SLE patients, serum RvD1 levels were reported to be lower than those in HCs [12,14]. The notable increase in RvD1 in patients with GBS may reflect a more effective resolution of inflammation, possibly contributing to the better prognosis observed in patients with GBS. Further research is needed to establish the causal relationship between RvD1 and GBS recovery and to elucidate the upstream mechanisms controlling differential RvD1 production between GBS and other neuroimmune disorders. Compared with currently available GBS biomarkers, RvD1 offers a more balanced profile of sensitivity and specificity. Ganglioside autoantibodies that target peripheral nervous system glycosphingolipids are highly specific biomarkers for GBS, but their sensitivity is limited: no single anti-ganglioside antibody is detected in most GBS patients [3,4]. Other serum biomarkers, such as low serum albumin, elevated neurofilament light chain, and serum cytokine alterations, are associated with GBS but lack disease specificity [3,4,20]. For example, the neurofilament light chain, a marker of neuronal damage, is also elevated in MS [20]. Taken together, these findings suggest that RvD1 complements these existing biomarkers by reflecting the activation of resolution pathways, thereby providing novel insights into the disease course and recovery potential.

Additionally, the dynamic changes in RvD1 during disease progression appear to be unique to GBS. The serum RvD1 level increased as the GBS advanced and was positively correlated with disease severity. Notably, RvD1 levels were significantly elevated in ventilation-dependent patients and in the axonal neuropathy subgroup, both of which are associated with greater disease severity. These results suggest that in GBS patients, RvD1 may be consistently upregulated to drive inflammation resolution. In severe cases, even higher RvD1 levels may be induced to counterbalance heightened autoimmune responses, thereby contributing to the self-limiting disease course of GBS. In contrast, autoimmune and neurodegenerative diseases with poor prognoses typically exhibit reduced RvD1 production, and RvD1 levels are negatively correlated with disease severity [18,21]. Compared with patients in remission, active SLE patients had lower RvD1 levels, and a negative correlation was observed between RvD1 levels and the SLE disease activity index [18]. Previous work has also demonstrated that the levels of several SPMs, including RvD1, decrease along the progression axis in AD [21]. Taken together, these data support the notion that the favorable clinical outcome of GBS may be attributed, at least in part, to adequate inflammation resolution. RvD1 may hold promise as a biomarker for assessing disease severity in patients with GBS.

In the EAN model, we observed overall upregulation of the RvD1 resolution axis—including RvD1 itself, its biosynthetic enzyme 12/15-LOX, and its receptor ALX/FPR2—in the blood, spleen, and sciatic nerves. Similar upregulation of SPM synthetase and receptors was reported in peripheral blood mononuclear cells from MS patients [19]. Considering that the majority of SPMs are reduced in MS, we speculate that although receptors for SPMs are upregulated, an insufficient supply of SPMs leads to impaired resolution signaling and unresolved inflammation [1,19]. These findings highlight the critical role of SPMs as active components in initiating resolution pathways.

Treatment with RvD1 promoted the resolution of inflammation in EAN, as evidenced by attenuated disease severity, amelioration of histopathological abnormalities, decreased serum pro-inflammatory cytokine secretion, increased anti-inflammatory polarization of T cells and macrophages, and increased apoptosis of T cells. These results suggest that elevated RvD1 levels in GBS patients may facilitate spontaneous recovery by driving inflammation resolution, thereby restoring homeostasis and creating a favorable foundation for subsequent tissue repair and functional recovery [[5], [6], [7]]. We further demonstrated that the therapeutic effects of RvD1 are translatable to EAE, highlighting the potential of inflammation resolution as a novel therapeutic strategy for autoimmune diseases. RvD1 possesses several unique advantages as an immunotherapeutic agent. First, RvD1 exerts multifaceted immunomodulatory effects on both innate and adaptive immune cells [[22], [23], [24], [25], [26], [27], [28], [29], [30], [31], [32]]. Our study demonstrated that RvD1 reprogrammed macrophages toward an anti-inflammatory phenotype and downregulated pro-inflammatory intracellular signaling. These findings are consistent with several previous studies in other disease models [[22], [23], [24]]. In addition to affecting macrophages, RvD1 also acts on other innate immune cells, such as inducing dendritic cell paralysis [25] and inhibiting neutrophil swarming [26], both of which play crucial roles in the pathogenesis of autoimmune diseases [27,28]. With respect to adaptive immunity, we found that RvD1 skewed CD4^+^ T-cell polarization toward anti-inflammatory subtypes and increased T-cell apoptosis, which are in line with earlier findings [18,29,30]. In addition, RvD1 reportedly reduces CD8^+^ T-cell infiltration [29,31] and modulates B-cell development, differentiation, and function [32]. Given the complexity of autoimmune pathogenesis, the use of RvD1 offers a broad-spectrum immunomodulatory approach in contrast to existing immunotherapies that primarily target single molecules or pathways. Second, RvD1 induces the resolution of inflammation without compromising immune defense [[5], [6], [7],^33^]. Unlike immunosuppressants such as glucocorticoids, which suppress both pathogenic and protective immune responses and are associated with adverse effects, including infection and malignancy, SPMs such as RvD1 reduce detrimental immune responses while preserving pro-homeostatic functions [[5], [6], [7]]. In our study, the phagocytic capacity of macrophages, which is crucial for the clearance of apoptotic cells and myelin debris, remained unaffected by RvD1 treatment. Previously, in microglia, the resident macrophages of the CNS, immune pathways downregulated by SPMs in AD remained largely unaltered under physiological conditions [33]. These findings suggest that RvD1 treatment preserves immune defense functions and may therefore be associated with fewer side effects than conventional immunosuppressants. Third, RvD1 promotes tissue regeneration, which is not observed with most current immunotherapies [[34], [35], [36]]. In a wound healing model, RvD1 enhanced the proliferation of periodontal ligament fibroblasts [34]; in an organ transplantation model, it supported bone regeneration via increasing osteoblast differentiation [35]; and in a muscle injury model, it facilitated skeletal muscle fiber regeneration by modulating muscle stem cell functions [36]. These regenerative effects represent a potential therapeutic advantage of RvD1. In our study, the RvD1 resolution axis was significantly upregulated in the sciatic nerve during the resolution stage, strongly suggesting its potential role in promoting tissue regeneration. Further studies are warranted to investigate whether RvD1 facilitates the process of remyelination in the context of GBS and MS.

The translational rationale of our study is grounded in a novel “disease-paradigm comparison” conceptual framework. In this context, we first identified RvD1 as a diagnostic biomarker in GBS, then validated its therapeutic effects in an EAN model, and finally, these effects were extended to EAE. This approach differs from existing strategies, such as “healthy-disease comparison” and “disease-disease analogy.” The healthy-disease comparison approach focuses on molecules that are altered in patients compared with healthy individuals, subsequently investigating their therapeutic potential [18]. The disease‒disease analogy approach extends therapeutic candidates from one disease to another on the basis of shared pathogenic mechanisms [37]. Against this backdrop, we propose a third strategy, the “disease-paradigm comparison” approach, which is grounded in both similarities and discrepancies between disease entities. In our study, this framework was applied to GBS and MS as “disease” and “paradigm”. While GBS and MS share several pathogenic commonalities [[1], [2], [3], [4]], their ultimate inflammatory fates diverge: GBS is typically self-limiting [3,4], whereas MS is characterized by chronic, unresolved inflammation [1]. This contrast provides a rationale for translating RvD1-related findings: in GBS, RvD1 appears to facilitate successful inflammation resolution, whereas in MS, insufficient resolution may contribute to disease persistence. Therefore, supplementing RvD1 in MS may help restore immune balance and promote tissue repair.

Our study has limitations and warrants further research. We observed some discrepancies between cohorts 1 and 2, such as differences in ROC performance and cytokine correlation patterns. These differences may result from batch effects, since sample collection and RvD1 detection for the two cohorts were performed independently. External validation in multicenter cohorts across diverse ethnic and geographic populations is needed to enhance the generalizability of our findings. A follow-up study is warranted to investigate longitudinal changes in RvD1 during convalescence, as well as its responsiveness to GBS treatment modalities, and to determine whether RvD1 may act as a prognostic biomarker for GBS. Validating the concentrations of RvD1 via liquid chromatography‒tandem mass spectrometry (LC‒MS/MS) would better support the findings.

At present, the research value of GBS and its animal model EAN may be underestimated. Owing to its relatively low incidence and self-limited disease course, GBS has attracted less scientific attention than other autoimmune disorders. However, the scientific question of why GBS typically has a favorable prognosis is of substantial scientific interest. Investigating the mechanisms of recovery in GBS patients could yield insights applicable to the treatment of other autoimmune diseases with poorer outcomes.

## Conclusion

This study elucidates the diagnostic and therapeutic potential of RvD1 in GBS and EAN. Compared with those in HCs and patients with other CNS diseases, serum RvD1 levels are elevated in patients with GBS, suggesting that RvD1 may serve as a diagnostic biomarker for GBS. RvD1 treatment ameliorated disease severity in both the EAN and EAE models, indicating its potential as a therapeutic agent for neuroimmune diseases. These findings offer new insights into the development of innovative treatment strategies for neuroimmune disorders.

## Compliance with Ethics Requirements

*All Institutional and National Guidelines for the care and use of animals (fisheries) were followed. All procedures followed were in accordance with the ethical standards of the responsible committee on human experimentation (institutional and national) and with the Helsinki Declaration of 1975, as revised in 2008 (5). Informed consent was obtained from all patients for being included in the study.*

## CRediT authorship contribution statement

**Ying Wang:** Conceptualization, Methodology, Investigation, Writing – original draft, Funding acquisition. **Shan Liu:** Conceptualization, Methodology, Investigation. **Mingqin Zhu:** Investigation, Writing – review & editing. **Qingxiang Zhang:** Investigation. **Hui Sun:** Investigation. **Kangding Liu:** Conceptualization. **Jie Zhu:** Conceptualization, Resources, Funding acquisition, Supervision, Writing – review & editing.

## Declaration of competing interest

The authors declare that they have no known competing financial interests or personal relationships that could have appeared to influence the work reported in this paper.
