# Supplementary material for: Diagnostic and therapeutic potential of resolvin D1 in Guillain–Barré syndrome
Source: J Adv Res. 2025 Nov 2;85:1085–98. doi: 10.1016/j.jare.2025.10.073 (PMC13316608; doi:10.1016/j.jare.2025.10.073)
Supplement: Supplementary Data 1 [file mmc4.docx]

Supplementary methods

**Histology and pathology of EAN sciatic nerve**

Mice were euthanized using 2% chloral hydrate and perfused with saline, followed by 4% paraformaldehyde. Bilateral sciatic nerves were harvested and post-fixed in 10% paraformaldehyde for 24 hours. The tissues were then dehydrated in a graded ethanol series, cleared in dimethylbenzene, and embedded in optimal cutting temperature compound. Sections of 4–6 μm thickness were obtained using a cryostat. To assess immune cell infiltration and neuronal demyelination, sections were stained with haematoxylin and eosin (H&E) and Luxol Fast Blue (LFB), respectively. Images were acquired using a laser confocal microscope (Nikon, Japan).

**Cytometric Bead Array assay**

Serum concentrations of anti-inflammatory cytokine interleukin (IL)-4 and IL-10, as well as pro-inflammatory cytokines interferon (IFN)-γ, IL-2, IL-6, IL-12, IL-17A and tumor necrosis factor (TNF)-α were determined using a Cytometric Bead Array assay (CBA), following the supplier’s instructions. In brief, samples and standards were incubated with a mixture of capture beads, and subsequently with PE-labelled detection reagents. Fluorescence for each cytokine was measured using a BD LSR Fortessa flow cytometer (BD Biosciences, USA) and analyzed using FlowJo software V.10. Cytokine concentrations were interpolated from standard curves.

**Assessment of RvD1 synthetase and receptor expression**

Mice were sacrificed at various stages of the disease. Peripheral blood was collected, and immune cells were isolated following erythrocyte lysis. Spleen and sciatic nerves were harvested and pulverised in liquid nitrogen. Total RNA was extracted using Mozol reagent according to the manufacturer’s instructions. RNA concentrations were determined at 260 nm using a spectrophotometer, and purity was assessed by the A260/A280 ratio. Only samples with ratios between 1.8 and 2.0 were used. One microgram of RNA was reverse-transcribed using MonScript™ 5X RTIII All-in-One Mix. Quantitative polymerase chain reaction (qPCR) was performed using MonAmp™ ChemoHS qPCR Mix to determine the expression of 12/15-LOX and ALX/FPR2, the synthesising enzyme and receptor of RvD1, respectively. Expression levels of 12/15-LOX and ALX/FPR2 were determined by qPCR using MonAmp ChemoHS qPCR mix using the following primers: 12/15-LOX-forward: 5'-GTCTACTCCACCACCTATTTTC-3', 12/15-LOX-reverse: 5'-CTGTGCTCATTGCCTTGTC-3'; ALX-FPR2-forward: 5'-TTCGTGAAACTGCACAAAGAG-3', ALX-FPR2-reverse: 5'-GCTCCATGACCTACTTCCTATC-3'; β-actin-forward: 5'-CCCACTCCTAAGAGGAGGATG-3', β-actin-reverse: 5'-AGGGAGACCAAAGCCTTCAT-3'. Ct values were obtained on ABI QuantStudio. Relative gene expression was calculated using the 2^−ΔΔCt^ method.

**Flow cytometry**

Flow cytometry was used to assess the phenotype and function of immune cells. Mice were sacrificed at various time points during disease progression, and spleens were collected. Following erythrocyte lysis and centrifugation, splenic immune cell suspensions were obtained. For T helper cell (Th) analysis, splenocytes were stimulated with leucocyte activation cocktail with GolgiPlug for 4 hours at 37 ^o^C before incubation with antibodies. For surface staining, cells were incubated with anti-mouse CD16/32 to block Fc receptors, followed by staining with fluorescently conjugated antibodies targeting surface markers: CD11b, CD206, CD25, CD4, CD40, and F4/80. For intracellular staining, cells were fixed and permeabilised, then incubated with antibodies against arginase-1 (Arg-1), Forkhead box protein P3 (FoxP3), IFN-γ, IL-4, IL-17A, and inducible nitric oxide synthase (iNOS). To assess the cell apoptosis, splenocytes were stained with Annexin V and 7-AAD. Unstained and isotype control samples were used to assess non-specific binding and cellular autofluorescence. Fluorescence was measured using a BD LSR Fortessa flow cytometer (BD Biosciences, USA), and data were analysed using FlowJo software (v10). The proportion of positive cells for each marker was defined as the percentage of cells with fluorescence intensity exceeding that of the isotype control.

**Assessment of macrophage phagocytosis capacity**

Mice were euthanized with 2% chloral hydrate, followed by intraperitoneal injection of 5 ml PBS containing 3% FBS. The peritoneal cavity was repeatedly lavaged for 15 minutes until the lavage fluid turned yellow. The lavage fluid was centrifuged, and the cell pellet was resuspended in culture medium.To induce phagocytosis, cells were incubated with 0.5 mg/ml FITC-pHrodo Green Zymosan A bioparticles at 37 °C for 30 minutes. After incubation, cells were blocked with anti-mouse CD16/32 and stained with anti-F4/80 to label macrophages. Phagocytic activity was evaluated by measuring fluorescence intensity using flow cytometry, and data were analyzed with FlowJo software (v10).

**Western Blot**

The expression of Toll-like receptor 4 (TLR4), MyD88, total and phosphorylated forms of p65, and β-actin in macrophages was assessed by Western blot. Splenic immune cells were isolated as described in section 2.9 and labelled with F4/80 microbeads. Macrophages were then purified using MACS columns according to the manufacturer’s instructions. Cells were lysed in RIPA buffer supplemented with 1% protease inhibitor and 1% phosphatase inhibitor. Protein concentrations were determined using the bicinchoninic acid assay. Equal amounts of protein were denatured and separated by SDS–PAGE, followed by transfer onto pre-activated PVDF membranes. Membranes were blocked, incubated with primary antibodies, washed, and then incubated with appropriate secondary antibodies. Signals were developed using HRP substrate and visualized using a chemiluminescent imaging system (SAGECREATION, China). Band intensities were quantified by calculating the ratio of target protein to β-actin using ImageJ software (NIH, Bethesda, USA).
