# Supplementary material for: Diagnostic and therapeutic potential of resolvin D1 in Guillain–Barré syndrome
Source: J Adv Res. 2025 Nov 2;85:1085–98. doi: 10.1016/j.jare.2025.10.073 (PMC13316608; doi:10.1016/j.jare.2025.10.073)
Supplement: Supplementary Data 2 [file mmc5.docx]

**Supplementary Table 1. Materials and sources**

| **REAGENT or RESOURCE** | **SOURCE** |
| --- | --- |
| **ELISA kits and Antibodies** |  |
| Resolvin D1 ELISA Kit | Cayman Chemical |
| Lipoxin A4 ELISA Kit | Cayman Chemical |
| Leukotriene B4 ELISA Kit | Cayman Chemical |
| Annexin A1 ELISA Kit | Cayman Chemical |
| Anti-mouse CD16/32 | BD Biosciences |
| Anti-mouse Arg-1 APC | BD Biosciences |
| Anti-mouse CD11b APC-Cy7 | BD Biosciences |
| Anti-mouse CD206 Qdot 585 | BD Biosciences |
| Anti-mouse CD25 Pacific Blue | BD Biosciences |
| Anti-mouse CD4 APC-H7 | BD Biosciences |
| Anti-mouse CD40 Pacific Blue | BD Biosciences |
| Anti-mouse F4/80 PE/Cy7 | BD Biosciences |
| Anti-mouse FoxP3 FITC | BD Biosciences |
| Anti-mouse IFN-γ PerCP-Cy5.5 | BD Biosciences |
| Anti-mouse IL-4 APC | BD Biosciences |
| Anti-mouse IL-17A PE | BD Biosciences |
| Anti-mouse iNOS PE | BD Biosciences |
| Rabbit-anti-Beta Actin | BD Biosciences |
| Rabbit-anti-TLR4 | Abcam |
| Rabbit-anti-MyD88 | Abcam |
| Rabbit-anti-p65 | Abcam |
| Rabbit-anti-p-p65 | Abcam |
| Goat Anti-Rabbit IgG (H&L) HRP | Abcam |
| **Chemicals and peptides** | |
| Resolvin D1 | Cayman |
| P0 peptide 180-199 | Multisyntech |
| MOG peptide 35-55 | GenScript |
| Mycobacterium tuberculosis | BD Difco |
| Incomplete Freund’s adjuvant | Sigma |
| Pertussis toxin | Merck Millipore |
| Isoflurane | RWD Life Science |
| Paraformaldehyde | Meilunbio |
| Phosphate buffered saline | Meilunbio |
| Hematoxylin and Eosin Staining Kit | Beyotime Biotechnology |
| Luxol Fast Blue Stain Solution | Solarbio |
| Protein marker | Thermo Fisher Scientific |
| Immobilon Western HRP Substrate | millipore |
| Tween-20 | Solarbio |
| RNase-Free ddH_2_O | Beyotime Biotechnology |
| MonScrip 5XRTIII All-in-one Mix | Monad |
| MonAmp ChemoHS qPCR mix | Monad |
| Monzol Reagent | Monad |
| Normal goat serum | Solarbio |
| Erythrocyte lysis buffer | BD Biosciences |
| Leuko Act Cktl With GolgiPlug | BD Biosciences |
| Fixation/Permeabilization Solution | BD Biosciences |
| Enhanced Cell Counting Kit-8 | Beyotime Biotechnology |
| pHrodo Green Zymosan A BioParticles Conjugate | Thermo Fisher Scientific |
| **Experimental models: organisms/strains** |  |
| C57BL/6J mice | Charles river |

Arg-1 = arginase-1; ELISA = enzyme-linked immunosorbent assay; FoxP3 = Forkhead box protein P3; HRP = horseradish peroxidase; IFN = interferon; IL = interleukin; iNOS = inducible nitric oxide synthase; MOG = myelin oligodendrocyte glycoprotein; PCR = polymerase chain reaction; TLR = Toll-like receptor.

Supplementary Table 2. Inclusion and exclusion criteria for the enrolled participants

| **Groups** | **Criteria** | |
| --- | --- | --- |
| **Common Criteria** | | |
| GBS, MS, NMOSD, and stroke | Inclusion | (a) Aged 18 years or older  (b) Currently in the acute phase of the disease |
|  | Exclusion | (a) More than 7 days after onset  (b) Coexisting other neurological disorders, autoimmune diseases, severe infections, or malignant tumors  (c) Severe chronic conditions such as, but not limited to, stage IV heart failure or dialysis-dependent renal disease  (d) History of immunotherapy within the past three months  (e) Any other factor known to significantly affect immune function |
| **Group-specific Criteria** | | |
| GBS | Inclusion | Fulfilled modified NINDS criteria for GBS [1] |
|  | Exclusion | (a) Diagnosed with Miller–Fisher syndrome  (b) Diagnosed with chronic inflammatory demyelinating polyradiculoneuropathy |
| MS | Inclusion | Fulfilled McDonald criteria for relapsing-remitting MS [2] |
|  | Exclusion | (a) Diagnosed with primary progressive MS  (b) Diagnosed with secondary progressive MS |
| NMOSD | Inclusion | (a) Fulfilled IPND criteria for NMOSD [3]  (b) Anti-aquaporin 4 IgG positive in serum |
|  | Exclusion | N/A |
| Stroke | Inclusion | Diagnosed with ischemic stroke |
|  | Exclusion | Without imageological supports |
| HC | Inclusion | (a) Aged 18 years or older  (b) No significant medical history |
|  | Exclusion | Any factor known to significantly affect immune function |

GBS = Guillain–Barré syndrome; IgG = immunoglobulin G; IPND = International Panel for NMO Diagnosis; MS = multiple sclerosis; N/A = not applicable; NINDS = the US National Institute of Neurological Disorders and Stroke; NMOSD = neuromyelitis optica spectrum disorders.

**References**

1 A.K. Asbury, D.R. Cornblath, Assessment of current diagnostic criteria for Guillain–Barre syndrome, Ann. Neurol. 27 (1990) 21-24. doi:10.1002/ana.410270707.

2. A.J. Thompson, B.L. Banwell, E. Barkhof, W.M. Carroll, T. Coetzee, G. Comi, et al., Diagnosis of multiple sclerosis: 2017 revisions of the McDonald criteria, Lancet. Neurol. 17 (2018) 162-173. doi:10.1016/S1474-4422(17)30470-2.

3. D.M. Wingerchuk, B. Banwell, J.L. Bennett, P. Cabre, W. Carroll, T. Chitnis, International Panel for, N. M. O. D. . International consensus diagnostic criteria for neuromyelitis optica spectrum disorders, Neurology. 85 (2015) 177-189. doi:10.1212/WNL.0000000000001729.

Supplementary Table 3. Assessment of disease severity in GBS, EAN and EAE

| **Disease conditions** | **Scoring system** |
| --- | --- |
| GBS | The GBS disability scale:  0 = healthy state  1 = minor symptoms and capable of running  2 = able to walk 5 m or more without assistance but unable to run  3 = able to walk 5 m or more with assistance  4 = bedridden or chair-bound  5 = requiring assisted ventilation for at least part of the day  6 = dead |
| EAN | 0 = no clinical signs  1 = paralyzed tail  2 = mild hind limb paresis  3 = moderate hind limbs paralysis  4 = severe hind limbs paralysis  5 = forelimb paralysis  6 = death  Intermediate symptoms were scored as 0.5 |
| EAE | 0 = no clinical signs  0.5 = partially limp tail  1 = fully paralysed tail  2 = titubation or hind limb paresis  2.5 = one hind limb paralyzed  3 = both hind limbs paralyzed  3.5 = hind limbs paralyzed with forelimb weakness  4 = forelimb paralysis  5 = moribund or dead |

EAE = experimental autoimmune encephalomyelitis ; EAN = experimental autoimmune neuritis; GBS = Guillain–Barré syndrome.

Supplementary Table 4. The optimal cutoffs for ROC curves

| Cohorts & groups | Cutoff^a^ | Sensitivity | 95% CI | Specificity | 95% CI |
| --- | --- | --- | --- | --- | --- |
| Cohort 1 |  |  |  |  |  |
| GBS vs HC | 112.7 | 0.81 | 0.60-0.92 | 0.76 | 0.58-0.88 |
| GBS vs MS/NMOSD | 76.33 | 0.71 | 0.57-0.82 | 0.86 | 0.65-0.95 |
| GBS vs stroke | 122.9 | 0.63 | 0.46-0.78 | 0.76 | 0.55-0.89 |
| Cohort 2 |  |  |  |  |  |
| GBS vs HC | 62.97 | 0.90 | 0.78-0.96 | 0.87 | 0.74-0.99 |

^a^RvD1 concentration (pg/ml)

CI = confidence interval; GBS = Guillain–Barré syndrome; HC = healthy control; MS = multiple sclerosis; NMOSD = neuromyelitis optica spectrum disorders; ROC = receiver operating characteristic RvD1 = Resolvin D1.

Supplementary Table 5. Cytokine concentrations at admission and progression stages in cohort 2

| **Cytokines^a^** | **Admission** | **Progression** | **Alterations^b^** | **P values^c^** |
| --- | --- | --- | --- | --- |
| IL-4 | 3.66 ± 0.31 | 6.40 ± 1.16 | 2.74 ± 1.17 | 0.026* |
| IL-10 | 2.14 ± 0.20 | 8.44 ± 1.58 | 6.30 ± 1.63 | <0.001*** |
| IFN-γ | 2.93 ± 0.29 | 4.66 ± 0.49 | 1.72 ± 0.57 | 0.005** |
| IL-17A | 28.79 ± 1.19 | 26.36 ± 3.15 | -2.43 ± 3.24 | 0.460 |
| IL-2 | 3.78 ± 0.53 | 3.89 ± 0.56 | 0.12 ± 0.54 | 0.829 |
| TNF-α | 2.12 ± 0.17 | 5.13 ± 0.813 | 3.00 ± 0.82 | <0.001*** |

^a^Concentration in pg/ml (mean ± SEM)

^b^Cytokine concentrations at the progression stage minus that at the admission

^c^Differences in cytokine levels between admission and progression groups

IFN = interferon; IL = interleukin; SEM = standard error of the mean; TNF = tumor necrosis factor.

**p* ≤ 0.05; ***p* < 0.01; ****p* < 0.001.
